# Maximizing Polyphenol Content to Uncork the Relationship Between Wine and Cancer

**DOI:** 10.3389/fnut.2019.00044

**Published:** 2019-04-30

**Authors:** Colin E. Champ, Anjali Kundu-Champ

**Affiliations:** ^1^Cancer Prevention Project, Pittsburgh, PA, United States; ^2^Department of Radiation Oncology, University of Pittsburgh Medical Center, Pittsburgh, PA, United States

**Keywords:** wine, cancer, phenolic, flavonoid content, radical oxygen species (ROS), biosynthesis of flavonoids

## Abstract

Studies have revealed conflicting results regarding the risk of cancer from alcohol consumption. Furthermore, some studies have suggested that wine may have benefits that separate it from other alcoholic beverages. As wine contains a significant amount of chemicals, specifically polyphenols like anthocyanins and proanthocyanidins (PA), that can affect cellular function and promote health, this hypothesis is reasonably supported by recent research. Polyphenols promote several anticancer cellular pathways, including xenobiotic metabolism, support of innate antioxidant production, and stimulation of phase I and II detoxification of carcinogens. However, the multitude of growing and production conditions of grapes, including temperature, water availability, soil type, maceration, and aging can result in a remarkably varying final product based on the available literature. Thus, we hypothesize that wines produced from grapes cultivated between steady daily temperatures at 15–25°C with moderate sun exposure from flowering to harvest, lower vine-water status, resulting either from lower precipitation, and irrigation practices or more permeable soil types, limitation of fertilizers, extended maceration, and aging in oak will impact the concentration of anthocyanins and PA in the finished wine and may have a differential impact on cancer. This higher concentration of polyphenols would, in theory, create a healthier wine, thus explaining the conflicting reports on the benefits or harms of wine.

## Introduction

With data emerging revealing the health benefits of a Mediterranean eating pattern, comprised of polyphenol-rich foods like olive oil and red wine, the impact of wine on overall health has been of great interest. Furthermore, according to the Organization of Vine an Wine (OIV), an intergovernmental organization overseeing the scientific and technical aspects of wine viticulture and viniculture, total global consumption of wine is staggering with nearly 645 million gallons consumed in 2017 alone (1). Wine is an important part of many cultures around the world, including Italy, Spain, and France, which are the world's largest producers, and in 2015, they collectively produced 48% of the world's total wine volume (2). In the United States, just over 50% of the population reports that they are routine consumers of alcohol (3). Since 1998, wine consumption in the United States has steadily increased, to an average consumption of 2.95 gallons/year/resident in 2016, reflecting a collective consumption of 3.6 billion total gallons of wine. While according to the World Health Organization 17% of alcohol consumed by Americans is wine, two thirds of alcohol consumption in European countries, such as Italy, is comprised of wine.

The risks and benefits of moderate red wine and alcohol consumption remain controversial. Recent meta-analyses have produced mixed results, revealing small increases in breast cancer in women and colon cancer in men, and small decreases in thyroid, lung, and hematologic malignancies (4). The majority of studies reveal increased cancer with excessive alcohol exposure, especially for oropharyngeal and esophageal cancer. Yet, a recent analysis revealed a potential increase even with light alcohol consumption (5). Making conclusions from the available research is difficult, as nutritional population studies introduce variables that are difficult to account for and can influence findings, and populations consuming alcohol are heterogeneous (6). Meta-analyses can aid in estimating cancer risk, but are less able to reduce confounding. For instance, alcohol consumption is often linked to cigarette smoking, which is established to increase the risk of multiple other cancers (7). Furthermore, alcohol and tobacco smoke may have a synergistic effect on the development of cancer (8), and a large analysis of women revealed that alcohol-related risk of aerodigestive cancer was limited to those women who both smoked and consumed alcohol (9). Alcohol consumption is also linked with socioeconomic status and comorbid conditions, like anxiety and depression, further negatively impacting studies (10). Finally, other studies suggest no increased risk of cancer with alcohol usage at moderate levels (<30 g/day), and additional risks in those who consume above this amount are confounded by obesity and poor folate status (11).

However, some feel as though moderate wine consumption may have a different physiologic impact when compared to other sources of alcohol. Further analyses of several studies assessing alcohol consumption and cancer reveal a lack of association between wine consumption and cancer (9), and others hint at a protective effect. For instance, the Copenhagen City Heart Study revealed that wine drinkers, as opposed to other alcoholic beverage drinkers, experienced a reduction in death from both coronary heart disease and cancer (12). This study and others have revealed a controversial J-shaped relationship between wine consumption and cancer: increased risk at no and high consumption. A recent meta-analysis of 111 unique cohort studies revealed a 7% increased risk of colon cancer for each 10 g/day increase in alcohol intake (13). However, a meta-analysis of eight case-control and nine cohort studies from the same year, including over 12,000 cases of colorectal cancer, revealed no relationship between wine consumption and colorectal cancer (14). Furthermore, studies have shown an association between improved survival after colorectal cancer diagnosis and pre-diagnosis wine consumption (15).

Several tangible mechanisms to describe a potential carcinogenic effect of alcohol exist. Ethanol in wine and other alcoholic drinks is metabolized by the liver via the enzyme alcohol dehydrogenase to form acetaldehyde. Ethanol is also metabolized to aldehyde via microbes within the gastrointestinal tract, which can also increase intestinal permeability (16). Acetaldehyde, a toxic carcinogen, is responsible for the typical hangover symptoms that occur after excessive alcohol intake. Furthermore, while ethanol itself does not appear to be carcinogenic, *in vitro* studies reveal that acetaldehyde can generate reactive oxygen species (ROS), damage both DNA and proteins, and produce cellular mutations (17). These mechanisms may explain both the increase in risk of aerodigestive cancer and alcohol consumption and the synergistic effect of smoking and alcohol. Additionally, alcohol consumption can affect serum hormonal levels and increase estrogen, potentially leading to an increased risk of breast and endometrial cancer (18).

However, to our knowledge, all studies assess quantity of ethanol consumption within alcoholic drink type subgroupings. Recent analyses in other food types reveal that the quality of foods, separate from quantity, can elicit distinct physiologic and metabolic responses. For instance, feeding animals their natural food for nourishment while allowing them to graze can result in significantly different fatty acid profiles and anti-inflammatory content that can result in measurable differences within humans after their consumption (19). In wine production, growing conditions, viticultural practice, and maceration/fermentation techniques can have a significant impact upon the resulting wine, producing a product that varies considerably in chemical composition. Thus, it is reasonable to hypothesize that a similar effect may be seen in wine drinkers. This article proposes that several factors should be taken into consideration in future studies that assess the relationship between wine and cancer.

## The Potential Benefits of Moderate Wine Consumption and Impact of Polyphenols

**Figure 1:**
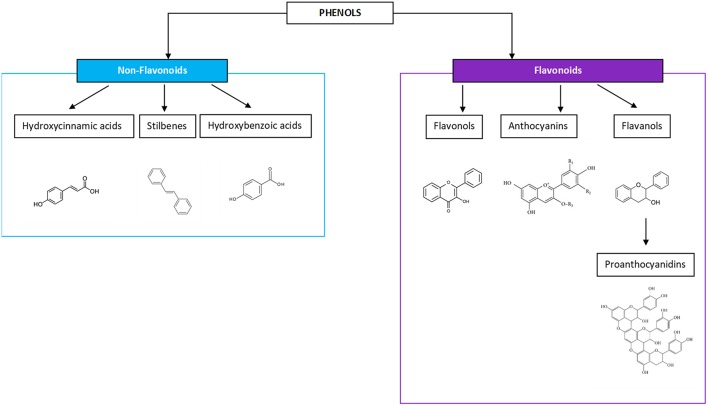
Phenols include non-flavonoid and flavonoid compounds, both of which are gaining more interest for the perceived health benefits.

Wine is a water-dominant solution containing a variety of chemical compounds, inclusive of aldehydes, esters, ketones, lipids, minerals, organic acids, phenolics, soluble proteins, sugars, and vitamins. In regards to the potential health benefits of wine, phenolics are considered the most important phytochemicals and have been given the greatest attention for their impact as an anti-oxidant constituent of wine, and for their ability to act as a free radical terminator and metal chelator (20). They possess many biological activities and health-promoting benefits. According to molecular structures, phenolic compounds exist in four major classifications: one phenolic ring (benzoic and cinnamic acids), two phenolic rings (stilbenes), three phenolic rings (anthocyanins, flavanols, and flavan-3-ols), and more complex ring structures (ellagic acids), the latter of which are found either in the Muscadine grape variety or more often are imparted on wines aged in oak (21–23). Flavonoids are the most dominant phenolics in a wine and come in the three-ring molecular structures—flavanols, anthocyanins, flavan-3-ols, and the oligomeric or polymeric condensed tannins, known as proanthocyanidins (PA), which are felt to be responsible for the antioxidant benefits from tea, fruit, and vegetable consumption (24, 25). The non-flavonoids present in three major types including hydroxycinnamic acids, stilbenoids, and phenolic acids and will not be the focus of this work, with the exception of vanillin, an aldehyde most prominent in the lignin structure of oak barrels (Figure 1).

Researchers have suggested that perhaps this combination of alcohol with certain substances or chemicals in wine may provide a benefit and explain the reduction in all-cause mortality (12). Preclinical data have revealed that these chemicals, and other pro-oxidants and antioxidants present in red wine in particular, may induce apoptosis in cancer cells (26). Furthermore, the consumption of red wine has a pro-oxidant effect that promotes the upregulation of human cellular antioxidant defense mechanisms, increasing serum antioxidant levels in humans, independent of vitamin C and carotenoids, similar to other phenolic-containing foods like strawberries and spinach (27, 28). These changes suggest the ability to offset potentially carcinogenic damage from ROS. *In vitro* and *in vivo* studies reveal a protective effect of wine polyphenols against oxidation and DNA damage from irradiation and hydrogen peroxide (29), animal studies reveal a reduction in DNA oxidative damage within the rat colonic mucosa after exposure to polyphenols and PA from wine (30), and human studies reveal increased serum total antioxidant status and decreased plasma glutathione and malondialdehyde after the consumption of wine, indicating a decrease in oxidative stress (31).

Recent studies reveal that polyphenols work via several mechanisms to promote anticancer cellular pathways. These pathways include xenobiotic metabolism, support of innate antioxidant production, and stimulation of phase I and II detoxification of carcinogens. These pathways are not necessarily activated via endogenous antioxidant properties of these chemicals, but rather via their pro-oxidant pathways that stress normal cells to activate nuclear factor erythroid 2-related factor 2 (Nrf2) and the human antioxidant response system (32). Nrf2 then triggers a plethora of genes that increase antioxidant production, reduce oxidative stress and oxidative damage, and activate the detoxification of potentially harmful chemicals (33).

This response is consistent with the role of polyphenols in grapes and other fruits and vegetables, as they serve as defense chemicals that are toxic to potential predators, including insects, animals, and microbials. When humans encounter these polyphenols, and other similar chemicals, these toxic effects do not occur, but rather the potential chemical threat elicits a cellular response leading to several chemopreventive changes. For instance, animal studies have revealed potential anti-carcinogenic and chemopreventive properties of the stilbene, resveratrol, via its activated phase II drug-metabolizing enzymes, inducing anti-inflammatory cellular function, and promoting antioxidant production (34). While these benefits are compelling, it is important to note that changes in polyphenol content in wine impact taste profile and sensorial characteristics of the wine, and the effect of this modification needs to be considered alongside consumer preference for various attributes of sugar, acid, and phenolic qualities of finished wine.

Bioavailability studies are generally difficult in practice, and thus limited in number, but reveal that polyphenols are absorbed via the lining of the intestines after modification by the epithelial cells in the small intestines and microflora within the colon (35). This process is variable, but can alter polyphenol content and bioavailability, before passage to the liver and entrance into the blood stream. While polyphenol structure and potency may be altered throughout the absorption process, studies reveal a subsequent increase in total plasma antioxidant status, and this is generally felt to be a direct result of the effect of the consumption of polyphenols. For instance, a study in healthy males revealed a significant loss in the expected rise of plasma antioxidant capacity after the consumption of phenol-stripped red wine (36). Red wine polyphenols have also been independently found to mediate the vascular effects of red wine consumption in humans, further supporting their ability to be absorbed (37). Lastly, urinary levels of 4-O-methylgallic acid, which are markers of phenolic acid absorption, are increased in humans after the consumption of red wine, and this absorption has been shown to offset lipid peroxidation in smokers (38).

Finally, chemicals released into the wine from wood during the aging process can significantly impact the chemical composition of the final product. For instance, Acutissimin A, a flavano-ellagitannin found in oak, can inhibit DNA topoisomerase II, a target for cancer (39). Furthermore, oak used for aging contains lignans, which are dimeric polyphenols exhibiting low estrogenic properties (40). Lignans are also present in tea, vegetables, and coffee, and have been shown to improve human health via several mechanisms. Lignans are metabolized by intestinal bacteria, promoting a healthy microflora (41). Data, however, are mixed, and some epidemiologic studies have suggested a potential protective effect in regards to breast cancer (42). While estrogenic themselves, lignans have been shown to reduce the production of estrogen, thus explaining this potential protective effect. In addition, a diet high in lignans through wine consumption has been associated with a reduced risk of the premalignant condition, Barrett's esophagus, and esophageal cancer (43, 44).

Here we investigate the impact of several known viticultural practices and the effect they have on synthesis of two critical phenols—anthocyanin and PA—in wine, postulating the importance of these elements and their antioxidant properties on human health, following consumption. Furthermore, we consider the environmental and viticultural processes that can increase their volume in finished wine.

## Anthocyanins and Proanthocyanidins

Anthocyanins and proanthocyanidins are secondary metabolites produced within the skins, seeds, and stems of grapes through the phenylpropanoid pathway and downstream flavonoid biosynthesis. These phenolic chemicals are produced as defensive and protective chemicals in response to environmental stresses, changing growing conditions, and plant predators, including herbivores and microbial and fungal threats. Other potential threats, including metals in the soil, can activate the production of both (45). Furthermore, they are also produced in response to ROS, and accordingly, superoxide radical scavenging activity correlates with polyphenol levels in the grapes (46). Anthocyanins are generally located along the epidermal surfaces of the growing plant, including the berry skin, and plant stems and leaves. Through their pigmentation properties, they serve as a protective barrier from excessive sun exposure, much like melanin in human skin. Anthocyanin accumulation within the berry typically begins at the onset of ripening, known as véraison. Anthocyanins represent a collection of several individual chemical constituents that have individual production peaks throughout the growing process, beginning with dehydroxylated glucosides (cyanidin, peonidin) followed by trihydroxylated anthocyanins (delphinidin, petunidin, malvidin) (47).

**Figure 2:**
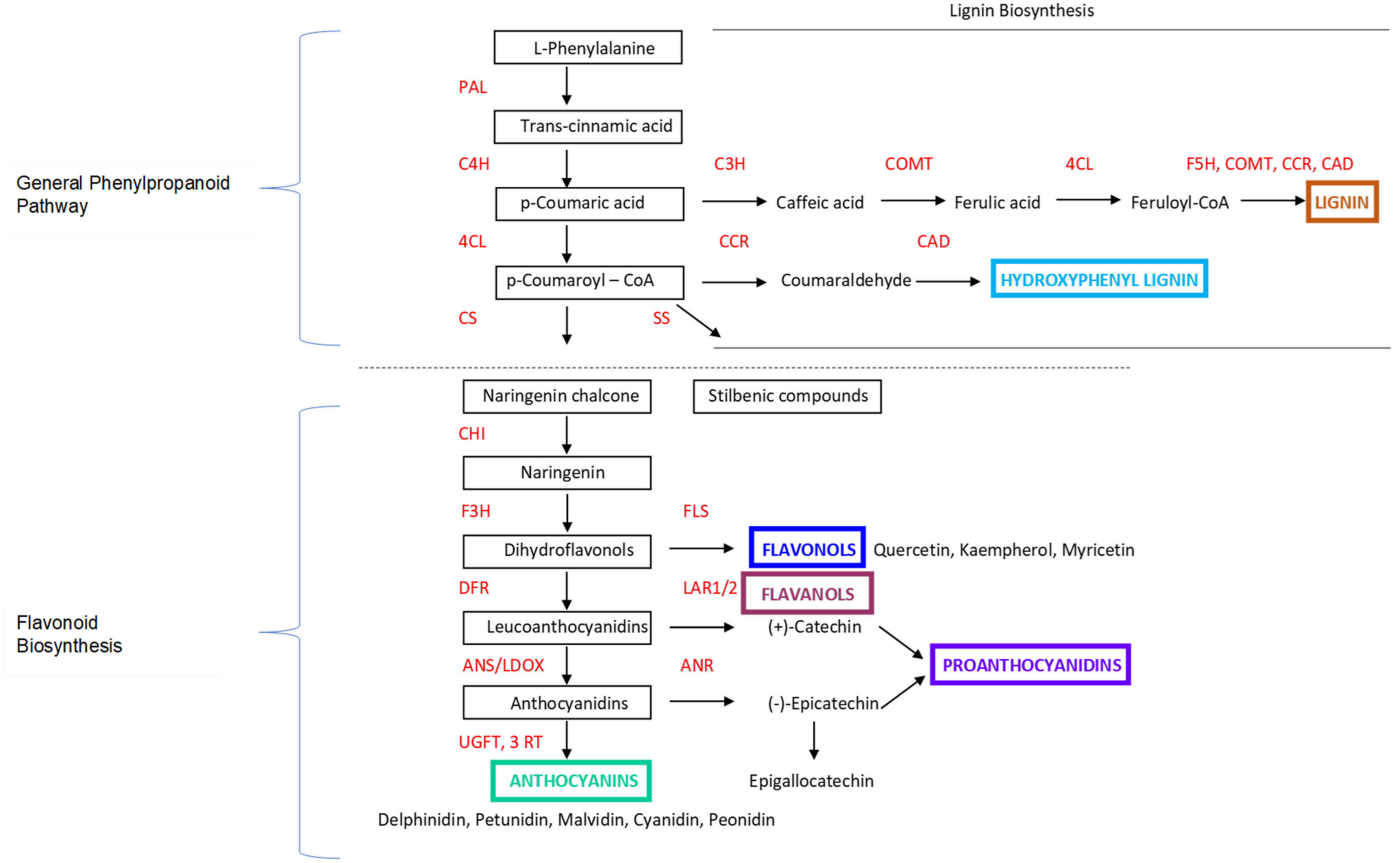
Marè et al. (48). Overview and simplification focusing on the key steps of the Phenylpropanoid Pathway and Flavonoid Biosynthesis in Grapes and Plants. PAL, phenylalanine ammonia-lyase; C4H, cinnamate 4-hydroxylase; 4CL, 4-coumarate-CoA ligase; CS, Chalcone Synthase; SS, Stilbene Synthase; CHI, chalcone isomerase; F3H, flavanone 3-hydroxylase; FLS, flavonol synthase; DFR, dihydroflavonol-4-reductase; LAR1/2, leucoanthocyanidin reductase 1/2; ANS/LDOX, anthocyanidin synthase/leucoanthocyanidin dioxygenase; ANR, anthocyanidin reductase; UFGT, UDP-glucose:flavonoid-3-O-glucosyltransferase; 3RT, anthocyanidin 3-glucoside rhamnosyltransferase. Additional information provided related to Lignin Biosynthesis. C3H, p-coumarate 3-hydroxylase; COMT, Caffeoyl CoA O-methyltransferase; 4CL, 4-coumarate:CoA ligase; F5H, COMT, CCR, CAD, Ferulate 5-hydroxylase; CCR, Cinnamoyl-CoA reductase; CAD, Cinnamyl alcohol dehydrogenase.

PA and flavonol compounds are generally found in the berry seeds and skins, as well as the stems. They follow a different biochemical and phonologic trajectory from anthocyanins (Figure 2), with their synthesis occurring at fruit set, between flowering and véraison; thereafter, oxidation and metabolism through ripening decreases the amount of PA until harvest (49, 50). These chemicals are a flavonoid plant secondary metabolite, functioning to deter herbivores and are characterized by antifungal properties and the deterrence of pathogens. They can also react with anthocyanins to stabilize them and aid in pigmentation (51). (+)-Catechin, (–)-epicatechin, and epigallocathechin flavan-3-ols are synthesized through the same flavonoid pathway as anthocyanins, but via a different regulation and enzymatic step (52), resulting in PA.

The concentration and composition of phenols, including anthocyanins and PA, is largely, although not exclusively, based on the grape cultivar, or variety (53). For instance, the presence of PA in Syrah, Cabernet Sauvignon, and Merlot is markedly higher than the concentration in Gamay, Pinot Noir, and Zinfandel (54), while Syrah, Malbec, and Cabernet Sauvignon have generally high concentrations of antioxidant-promoting phenols (55). The Tannat grape varietal has exceedingly high PA and total anthocyanin content, producing a firm tasting, full-bodied wine with tannic structure (56, 57). In addition, environment and viticultural practice, along with harvest date, are other critical factors affecting accumulation of both anthocyanins and PA, along with acid and sugar profiles desired by consumers, and these factors in combination differentiate finished wine products and their related antioxidant capability (53).

Evidence is building to propose that elements of the flavonoids contribute to important bioactivities such as antioxidant, cardioprotective, anti-inflammation, anti-cancer and anti-aging mechanisms, garnering more interest and scientific evaluation (Table 1) (97, 98). For instance, anthocyanin isolates and anthocyanin-rich mixtures of bioflavonoids may provide protection from DNA cleavage, estrogenic activity (altering development of hormone-dependent disease symptoms), enzyme inhibition, boosting production of cytokines and regulating immune responses, anti-inflammatory activity, lipid peroxidation, decreasing vascular permeability, and membrane strengthening (99). Meanwhile PA have been shown to suppress free radicals and potentiate other antioxidants such as vitamin C and vitamin E, while also strengthening capillaries in the mitigation of chronic venous insufficiency, lowering blood pressure, slowing the progression of diabetic retinopathy, and increasing the amount of UV rays necessary to cause sunburn, reducing photocarcinogenesis (100). PA, predominantly those found in grape seeds, have also been shown to have anti-carcinogenic activity on tumor models related to prostate, metastatic breast and colorectal cancer (101, 102).

**Table 1:** Selected phenolics common in red wines and their associated biological activities and health-inducing effects.

**Phenol class**	**Sample biological activities and health protective attributes**	**References**
**NON-FLAVONOIDS**		
Hydroxycinnamic Acids	Prevention of oxidative stress, diabetes, insulin resistance, body weight gain, dyslipidemia, cardiovascular disease, renal disfunction Enhanced potency as an anti-inflammatory agent Improvement of liver function	(58, 59)
Stilbenoids	Cancer treatment and prevention, through mechanisms of apoptosis, inhibition of angiogenesis and proliferation of multiple cancer cell lines Neuroprotection, through reduction of amyloid plaques, cerebral infarct volume, neuronal ROS, and inhibition of cholinesterases Depigmentation, through decreased melanin production and inhibition of tyrosinase activity Protection against cardiomyocyte and cardiac hypertrophy, through activation of AMPK and upregulation of eNOS Blood Pressure Regulation, through lowering of systolic blood pressure at high doses Reduced Platelet Aggregation, through inhibition of COX enzymes Reduction in Obesity, through inhibition of lipogenesis, increased lipolysis, activation of AMPK, SIRT and PGC-1α Management of Diabetes, through enhanced insulin sensitivity, increased AMPK-dependent microbial biogenesis, and increased glucose uptake Defense against Atherosclerosis, through reduction in oxidative stress markers and inhibition of LDL in endothelial cells Protection against UV radiation resulting in reduced DNA damage, skin damage and cancer Protection against Ischemia-reperfusion injury, through increased antioxidant enzymes and reduced oxidative stress	(60–64)
Hydrobenzoic Acids	Antimicrobial properties Amelioration of cardiovascular problems such as hypertension, atherosclerosis, and dyslipidemia Neuroprotection and anti-inflammatory effects providing defense again neurogenerative diseases of Alzheimer's and Parkinson's diseases and amyotrophic lateral sclerosis (ALS)	(65–67)
**FLAVONOIDS**		
**Flavonols**		
Quercetin	Induction of apoptosis, which has been particularly effective in decreasing growth of brain, liver and colon cancers Reduction of coronary artery disease due to increase flow-mediated dilation of major arteries Modulation of inflammation by way of COX and lipoxygenase inhibition Reduction of oxidative damage to lymphocytes and neurovascular structures which inhibits damage to neurons and protects against neurodegenerative disorders Gastroprotective effect by way of inhibition of gastric acid secretion Anti-allergic effect, through inhibition of release of histamine from mast cells	(68–70)
Kaempherol	In combination with Sora, inhibits cell growth inhibition in the S-phase and/or G2/M-phase for hepatocellular carcinomas Induction of cell cycle arrest in proliferating cells Impairment of cancer angiogenesis through inhibition of VEGF secretion in cancer cell lines Inhibition of MMP-3 protein activity in highly-invasive MDA-MB-231 breast cancer, reducing tumor invasion Interruption of cellular pathways (TNF-α, JAK3, IL-8, IL1B) responsible for inflammation	(71–76)
Myricetin	Cytotoxicity in many cancer cell lines including hepatic, skin, pancreatic, and colon cancers Anti-inflammatory effects on periodontitis and rheumatoid arthritis, through various cellular mechanisms Protective effects against Parkinson's Disease and Alzheimer's Disease Vascularprotective effect, through alteration of vascular disease-related genes, including HIRA, HDAC9, HIF1A and RTN3 Management of non-insulin-dependent diabetes, by stimulating the uptake of glucose without functional insulin receptors	(77–79)
**Anthocyanins**		
Delphinidin	Inhibition of EGFR and downstream signaling cascades, most notably in vulvar carcinoma Decrease in cell viability, induction of apoptosis, cleavage of PARP, activation of caspases−3,−8, and−9, increase in Bax with concurrent decrease in Bcl-2 protein, and cell cycle arrest in G2/M phase, in colon cancer Mediated suppression of osteoclast formation, resulting in prevention of bone loss Increase in insulin secretion	(80–83)
Petunidin	Inhibition of breast cancer and liver cancer cell growth, via inhibitory behavior Increase in insulin secretion Inhibition of alpha-glucosidase and lipase in alleviation of diabetes	(83, 84)
Malvidin	Inhibition of various tumor cell lines including promyelocytic/monocytic leukemia cells, colon cancer cells, and gastric adenocarcinoma cells Increase in insulin secretion Antihypertensive activity by inhibition of angiotensin I-converting enzyme and anti-inflammatory effect by blocking NF-κB pathway Counteractive effect on oxidative stress in neuronal cells	(83, 85, 86)
Cyanidin	Inhibition of EGFR and downstream signaling cascades, most notably in vulva carcinoma Induction of cancer cell apoptosis, reduced oxidative damage to DNA, inhibited cell growth and decreased cancer cell proliferation, notably in leukemia, lung, colon, skin and prostate cancer Prevention of Type II Diabetes through enhanced adiponectin and leptin secretion Preventative effect on Type II Diabetes through increased phosphorylation of AMPKα at Thr172 in rat adipose cells	(80, 87)
Peonidin	Chemopreventative and anti-inflammatory effect on inhibition of TPA-induced COX-2 expression, and decreased TPA-induced neoplastic transformation and blocked TPA-induced phosphorylation of extracellular signal–related kinases in breast cancer cells	(88)
**Flavanols**		
(+)-Catechin	Mediation in cardiovascular health via mechanisms of blood pressure reduction, flow-mediated vasodilation, and atherosclerosis attenuation Induction of antioxidative and neuronal anti-inflammatory effects in Alzheimer's Disease	(89, 90)
(–)-Epicatechin	Improvement of endothelium-dependent flow-mediated dilation of the brachial artery important for vascular health Attenuation of atherosclerosis via modulation of NF-κB activity	(91)
Proanthocyanidins (oligomeric/polymeric)	Anti-tumor activity in PC-3 prostate cancer cell lines, through cell cycle arrest, and activation of caspase-3 Inhibition of NF-κB and downstream cascades in human epidermoid carcinoma Reduction in proliferation, increased apoptosis, cell cycle arrest and modulation of key genes beneficially regulates invasion and metastasis for prostate and photocarcinogenesis Protection against oxidative stress and degenerative diseases including cardiovascular dysfunctions, acute and chronic stress, gastrointestinal distress, neurological disorders, pancreatitis, various stages of neoplastic processes, and carcinogenesis including detoxification of carcinogenic metabolites Reduction in hypertension via inhibition of the reactive oxygen species/mitogen-activated protein kinase pathway via restraining the release of ET-1 Inhibition of lipid peroxidation, platelet aggregation, capillary permeability and fragility, and to affect enzyme systems including phospholipase A2, cyclooxygenase, and lipoxygenase	(92–96)

## Environmental Conditions

“Terroir” is the French term used to define the geographical and environmental position of grapes. Terroir consists of several environmental factors that can influence its phenotypic expression, including geography, geology, and vertical zonality or altitude in which a vine is grown (103). Terroir has a major influence on the taste profile and typicity of the wine, but more importantly for its effect on grape composition, including phenolic content, whereby it creates a differential wine that can influence health. The following are several aspects of terroir considered in their influence on anthocyanin and PA accumulation.

### Temperature and Sun Exposure

Temperature and sun exposure are significant factors in grape growth and finished wine. They are generally considered together as they have shared responsibility in activation of the phenylpropanoid pathway and synthesis of anthocyanins and PA. Both appear to result in a similar effect on plant biosynthesis of these chemicals and separating the effect of sunshine vs. warmth is difficult, except in *in situ* studies (104–106).

Temperature is one of the primary elements of *terroir*. While grapevines are grown in a wide variety of climates, the major wine-growing regions in the northern hemisphere require adequate warmth without excessive heat, and thus are located between the 35th−50th parallels and in the southern hemisphere, are located between the 30th−45th parallels. The Mediterranean climate regions, known for their warm and dry summers and cool and wet winters, meet similar criteria, are often considered to be the optimal environment for viticulture, whereas, tropical climates do not serve as compatible environments for grapevine growing due to excessive heat, precipitation, and humidity (104).

Moderate temperatures during the night and day (15–25°C, respectively) promote the biosynthesis and accumulation of anthocyanins in the skins of the grape berries, while temperatures exceeding 35°C can decrease the accumulation of these chemicals through both the inhibition of biosynthesis pathways (107). and via the degradation of anthocyanins in grape skins (108, 109). Elevated and excessive temperatures can shorten phenological stages and impact bud break, flowering, véraison, ripening, maturity, and harvest, and affect wine quality. Elevated temperatures at night appear to affect anthocyanin accumulation and profile via the inhibition of anthocyanin mRNA transcription (110), but only during the véraison period (111). Conversely, low nightly temperature can enhance anthocyanin production (112).

Sunlight exposure is influential in the level of anthocyanins present in grape berry skins, as ultraviolet light is a fundamental prerequisite for color formation in grapes and other plants (109). Anthocyanins are produced as a method to provide protection against solar radiation and serve to protect photosynthetic tissues from ultraviolet damage, absorbing blue-green, and ultraviolet light. Transcription and expression of genes such as UDP-glucoside: flavonoid glucosyltransferase (UGFT) is induced by light, which has the net effect on the cellular mechanisms aiding in anthocyanin synthesis (113). Many studies evaluate the sunlight exposure and microclimate of various cultivars, whereby grape berries move through their phenologic phases under naturally occurring conditions, with application of berry cluster thinning, removal of basal leaves, and with canopy shading regimens (114). This manipulation of the microclimate, of the localized environment created naturally by the grapevine itself, has garnered great interest from a viticultural perspective, as intentional adjustments on the plant itself can regulate sun exposure and optimize anthocyanin production.

Studies conducted in merlot and carmenere berries and skins conclude that temperature has a non-significant or secondary effect on PA accumulation; although there is a direct relationship between berry mass and PA content, along with a direct relationship between temperature on berry growth (115). Other studies reveal a complex relationship between temperature and PA production that relies on multiple factors, including season, vine age, and grape type, among others (52). Further research is needed to elucidate this relationship.

While temperature has a poorly understood or non-significant effect on PA, sun exposure has a more recognizable impact on PA accumulation, notably signaling an increase in PA production in grape skins from flowering to véraison (52). A number of studies have been conducted on various cultivars including Merlot, Shiraz, and Pinot Noir, which have yielded somewhat conflicting results. Lee et al. revealed no significant difference in PA content of light-excluded and non-light-excluded Merlot samples, consistent with the findings of Downey et al. (116). However, (117) found a result of higher PA concentration in light-excluded Shiraz, while Cortell found that PA were lower in light-excluded Pinot Noir. Several reasons explain these conflicting results, namely, analytical method, variations in growing vineyards and notably cultivar, as is elucidated by Asproudi et al. (118) and Lee (119).

### Water Stress

Plant growth and production has historically been associated with geographies where rainfall is sufficiently supportive for growth, while avoiding excessive rain and overwatering. Along these lines, intentional water restriction can have significant effects on the chemical profile of grape varietals. Targeted water deficits and regulated irrigation can provide the grape vine with water stress and maximize the production and concentration of anthocyanins and PA, and this effect is more pronounced in red in comparison with white cultivars, which naturally have lower anthocyanins and PA volumes (120). Precipitation along with water deficits, both their amount and timing (phenological stage), affect vegetative growth, berry growth, fruit quality, titratable acidity/malic acid, phenolics, and yield.

While precipitation, in moderation, is essential for most plant life, extended periods of rainfall are specifically associated with increased risk of diseases in *V. vinifera*, including downy mildew and *botrytis* rots (121). Further, direct rain damage to ripening berries results from rapid swelling from excess water intake, which increases osmotic pressure and can hinder biosynthesis of phenols (122). Temperatures up to 25°C accompanied by low rainfall, induce the maximal concentration of polyphenols in grapes (123). Supporting these findings, research has shown that when Cabernet Sauvignon experiences lower rainfall in its dormant stage, vines produce looser clusters, heavier berry skins and higher total soluble solids (124). In comparing two vintages, the drier vintage had lower measurable vine water status and lower Nitrogen levels in the soil, and it had a higher resulting concentration of anthocyanins.

Thermal and water stresses can also synergistically enhance grape polyphenol production, while higher irrigation dose (patterns of watering and re-watering) reduces phenolic content of finished wines (123). These results have been substantiated by several studies, which found specifically that water stress during pre-véraison (onset of ripening) stages has the ability to ignite metabolic changes within the grape that remain after re-watering, activating, and altering transcripts and metabolites involved in phenyl propanoid, isoprenoid, carotenoid, amino acid, and fatty acid metabolism (120). Moreover, lower vine water status results in smaller berries, but thicker and heavier skin weights, correlating to higher anthocyanin concentrations, illustrating the sensitivity of anthocyanin biosynthesis to osmotic stress (124). Studies in Merlot, Shiraz, and Cabernet Sauvignon cultivars all reveal a correlation between water deficit and increased expression of genes responsible for anthocyanin synthesis, and the resulting accumulation of anythocyanins (125, 126). When assessing four stages of phenologic development, including pea size, véraison, maturation and full maturation, researchers observed that under sustained deficit irrigation, PA increased (127). Furthermore, at full maturation, anthocyanin and PA content is significantly higher in the skins and seeds, respectively, treated with both sustained and regulated deficit irrigation as compared with non-irrigated berries (128).

### Soil Type and Fertilizers

Vineyard geology—bedrock and overlying soils—is widely considered to help explain the typicity of wine from a particular region. Soil types may have an impact on the composition of finished wines, just as soil has an impact on any agricultural product due to mineral uptake. Grapes are vinified in a variety of soil conditions including igneous, metamorphic, sedimentary and textured soils, all with varying degrees of density, penetrability, and mineral content. Expectedly, soil type influences water content and irrigation as previously discussed, along with ground temperature, thus influencing grape growing and the resulting agricultural product, including wine. However, to our knowledge, limited attention has been given to soil type as a standalone factor—perhaps because it is difficult to isolate and control this in the presence of pH and mineral composition—and any independent effect it may have on phenolic compounds in grape berries.

An assessment of Cabernet Sauvignon grown in the Helan Mountain range in Ningxia, China, found that grapes grown in eolian soil (loess) and sierozem had increased anthocyanins and more phenols, as compared with irrigation silting soil (129). Sierozem soils are characterized by good physiologic properties, high biological activity and fertility resulting in high yields. Eolian soil, which consists of sand and sediment, performs similarly to rocky soils by limiting water retention given the ease with which water drains through the soil crevices.

The application of fertilizer can influence the final wine product and its chemical composition. Fertilization is most often utilized when the soil itself cannot provide sufficient nutrients to the plant to improve or maximize yield. In *V. Vinifera* the most common fertilizer additives in vineyards include treatments with nitrogen, potassium, and phosphate. The former two have been more extensively studied and have been demonstrated to reduce the color of grapes under higher application doses (130–132).

Nitrogen, in combination with other environmental influences, can lead to excessive plant growth, known as vigor, but can decrease the overall yield of berries (133). Grapevines which exhibit excessive vigor have excessive shoots, triggering an imbalance between the relative amount of vegetation and fruit, with vegetation prevailing. Excessive vigor leads to shaded fruits, which results in reduced activity of the photosensitive steps and MYB-family gene regulatory processes involved in the flavonoid biosynthesis occurring in the phenylalanine pathway, resulting in lower anthocyanin synthesis (130). Essentially, nitrogen-induced grapevine vigor can act as a barrier to reduce the amount of light in contact with the berries within the canopy, reducing the activity of photosensitive enzymes that regulate the phenylpropanoid pathway and limit production of phenolic compounds, namely anthocyanins (134). Importantly, nitrogen acts in different ways in different soil types. In general, in soils with lower organic matter and/or nitrogen, low nitrogen stress can enhance the production of anthocyanins (135).

Potassium plays a crucial role in biotic and abiotic stress response, plant physiology and biochemical processes such as photosynthesis, osmoregulation and enzyme activation (136). In grape berries, potassium is found in high concentrations when measured at harvest, accounting for nearly 80% of all the cations within a grape berry (137). Grapes and other plants produce potassium as a response to several stresses, including water stress and insect predators. Low potassium, or potassium stress, can result in the accumulation of ROS within the plant, which prompts the production of antioxidant defense mechanisms and polyphenols to scavenge free radicals, leaving them more acclimated for enhanced survival (138). The application of potassium in soil decreases insect infestation or other pathogenic attack, encouraging a strong cell wall and stimulating phenol production to reduce vulnerability and prevent further infection (139, 140). In the grape berries, it also aids in the balance of pH and acidity in finished wine. However, like nitrogen, concentration of potassium varies with soil type and its chemical and physical properties. Soils naturally rich in potassium, such as is the case in many Australian wine regions, often require manipulation to produce artificial potassium stress (141, 142).

Metals present in the soil can impart toxicity to the growing vines and stress the grape plant, prompting the production of antioxidant metabolites and polyphenols within the plant (143). Presence of Sr, Mn, Si, and Ph content in soil was shown to lead to higher concentration of antioxidants in grapes (45). Polyphenols aid in plant defense of potentially toxic metals by acting as chelators, and free-radical-induced stress via peroxidases enhances metal chelation, linking the multiple defense mechanisms of the growing grape vine (144).

Biostimulants should also be briefly addressed, due to their impact on grape growth. They are defined as substances able to alter physiologic plant processes that result in benefits in enhanced nutrient uptake, growth, development, yield and response to abiotic stress which improve quality (145). Several categories of biostimulants exist, including, bacteria, fungi, seaweeds, higher plants, animals and humate-containing raw materials, among others (146). Biostimulants can be introduced via foliar application to the grapevine and have been shown to positively affect phenylalanine ammonia lyase activity and gene expression within the phenylpropanoid pathway, resulting in greater primary and secondary metabolism of phenolic compounds (147, 148).

Overall, small amounts of nutrients are required within the soil for optimal grape growth and maturation. However, excessive nutrients, much like excessive water and heat, can hinder anthocyanin and phenolic production by alleviating required plant stress. Grape vines that are stressed with low, but adequate levels of nutrients and minerals appear to produce the largest accumulation of anthocyanins and phenolic chemicals. Fortunately, through viticultural and field practice, winemakers can adjust and regulate mineral elements which can influence the production of these phenols.

### Maceration and Fermentation

Berry maturation at harvest, and the concentration of anthocyanins and PA can influence the final wine profile, in combination with extractability and stability through maceration and fermentation. Maceration is the process whereby the crushed grape, skins, seeds, and even stems remain in a vessel and are given adequate time for fermentation. Maceration is divided into three stages: pre-fermentation, fermentation, and post-fermentation. During the process, the phenolic materials of the grape—including anthocyanins and PA, and other phenols—are leached from the grape skins, seeds, and stems into the must. Of all aspects of grape growth and wine production, maceration has the most significant influence on anthocyanin and PA content of the wine. Interestingly, while viticultural methods can increase or decrease anthocyanin, PA and other phenolic volumes, most winemakers rely heavily on maceration to make corrections or adjustments to their finished wine product. Extraction of phenols from the must is dependent on time of contact, temperature of maceration, and ethanol levels during fermentation (53, 149).

Maceration is a method of condensing and concentrating the volume of phenols in wine and is most commonly associated with red wine. Red wine derives its darker red color from phenolic and pigmented chemicals leached from the grape parts. White wine maceration is considerably shorter than red wine, limiting exposure and contact with the skin, seeds and stems, and thus leaves an un-pigmented wine (150, 151). Though, “orange wines” or skin-contact whites are beginning to come into wider production, namely in the Veneto Region of Italy, and also in Slovenia.

Anthocyanins are found mostly in the skin of grapes, imparting them with their blue, purple, and red color. When they are crushed and left to ferment in the winemaking vats, in general, the longer the juice is exposed to the skins and seeds, the more color is imparted on the wine due to chemical leaching (152). Anthocyanin extraction is in many cases considered easier than the extraction of PA during the maceration process (153). Several studies have shown that between days 3and 5 of maceration is when the majority of anthocyanins are diffused, inherently because of the water-soluble nature and accessible epidermal location of anthocyanins (154). Conversely, maceration times exceeding 20 days can reduce the volume of anthocyanins due to several mechanisms, including their absorption by yeast and also the rising level of ethanol (155, 156). However, PA and other phenolics continue to be secreted after this point (157).

PA concentration and extractability is greatly influenced by the cultivar (158). Skin PA are extracted more rapidly and readily than seed PA with studies revealing that two to three or more weeks of maceration and contact time result in the highest levels of PA extraction (157). Skin PA extraction reaches a plateau prior to pressing whereas seed PA increase progressively through the maceration phase (159, 160). Seed PA extraction is favored and accelerated by the presence of alcohol (157, 158, 161). Overall, extended maceration time appears to greatly influence PA content of wine. Skin contact of 3 to 4 weeks or longer appears to leach the largest amount of PA into wine; however, consideration of shorter maceration times for preservation of anthocyanins must also be considered (157, 162, 163).

Often winemakers apply temperature variations in the maceration process, which can affect the finished wine. The most commonly used methods are heating or cold soaking at the end of maceration. Heat treatment, like the addition of pectolytic enzymes, is intentionally applied to damage grape hypodermal cell walls and membranes to aid in extraction of phenolic compounds (164). Heat, in combination with aqueous ethanol increases this effect, until the point at which ethanol begins to boil (78.5°C), or it begins to oxidize or degrade the desired compounds (165, 166). Reports vary, with some showing an increase in anthocyanin levels via this method, but the majority reveal a destructive effect on the phenolic chemicals (167).

Cold soak treatment occurs when grape berries are mashed, cooled to a low temperature and then kept for several days. This is typically achieved by cooling the fermentation tank, placing the holding vessel in a cool, ambient environment, or by applying dry ice, characteristically achieving a temperature below 10°C (164, 165). In a study assessing several popular red varietals, including Cabernet Sauvignon, Malbec, and Merlot, the cold-soak technique enhanced the extraction of anthocyanins, but inhibited the extraction of PA, which with controlled application, could be valuable in balancing astringency and bitterness (168). Adjusting the duration of cold soak (0, 1, 4, 7, and 10 days) impacts the extraction of anthocyanins, determining that extraction increases in all cases at least initially, but beyond the 5th day of cold-soak treatment, anthocyanins begin to react with other compounds, thus lowering overall concentration (169). This effect, however, is dependent on the grape variety and its associated physical properties; Tannat grapes, for instance, have hardy cell walls and cold soaking combined with extended fermentation significantly increases anthocyanin content in wine produced from this grape variety (56).

During the fermentation process levels of sugar steadily decrease as the yeast metabolizes it, producing alcohol in the process (170). The presence or addition of sugar can be detrimental to anthocyanin levels, as it promotes its degradation (171). However, high levels of alcohol can degrade anthocyanins as well (172).

Finally, fining agents can be used for a number of reasons, including clarification, eradication of off-aromas, and reduction of bitterness or astringency to provide a softer wine and appeal to a wide audience of wine drinkers. Fining techniques include agents that can bind and filter certain components of the wine, and include egg albumen, casein, bentonite, elatine, skim milk, carbon, activated carbon, Isinglass, and Polyvinylpolypyrrolidone (PVPP) (173). While winemakers often rely of fining to stylize or achieve a desired sensorial affect, overutilization of fining can remove anthocyanins, PA and other phenols, often leaving a less pigmented wine (174). The minimization or avoidance of fining will generally yield wine with generally higher levels of polyphenols, enhancing its positive health influence.

### Wine Aging: (Wood Tannins)

Although not the focus of this discussion, wine aging, specifically in contact with wood, should be addressed due to its effect on the chemical composition of wines. Wine is often aged within wood barrels, or with the introduction of staves and chips. When barrels are utilized, wines are placed, most commonly, in American oak (*Q. Alba*) or French oak (*Q. robur, Q. petraea*) barrels to age and undergo gentle oxidation, which results in decreased astringency and concentrates color and stabilization of pigment structures (175). These elements allow soluble oak extracts to diffuse into the wine while contributing to sensorial characteristics within the wine. However, much like the skins, seeds, and stems of wine, wood used for aging has naturally occurring tannins that can leach into the aging wine. Of note, excessive aging in the bottle, on the other hand, can result in breakdown of anthocyanin. Thus younger wines would be expected to have elevated levels of phenolic chemicals (176).

Ellagitannins, a type of hydrolysable tannin, different from condensed tannins or PA in grapes, are concentrated in oak bark. As an extract into finished wine, they are associated with bitterness and astringency, a signal of their use in nature to deter potential predators (177).

The effect and degree of toasting of oak barrels, chips and staves also influences both organoleptic profiles and polyphenol content in wines. Toasting is a technique in which a barrel is fired, and in which wood tannins are mellowed and raw oak flavors are muted. Toasting is often considered at three temperature-regulated levels, including light (LT), medium (MT), and heavy (HT), and at these varying levels, wood lignans, hemicelluloses and cellulose structures undergo changes effecting permeability that ultimately influence the wines aged within them (178). LT yields the highest levels of ellagitannins. On the other hand, heavier toasting disrupts lignin bonds, which results in greater concentrations of vanillin, syringaldehyde, guaiacol, and furfurals, important for organoleptic properties of the wine, in addition to enhancing the abundance of beneficial compounds in finished wine. *In vivo* and *in vitro* studies show antitumor, anti-invasive, antimetastatic and antiangiogenic qualities of vanillin, and its therapeutic potential in cancer prevention and treatment is of continued interest in the research community (179).

Barrels from oak species are the most common cooperage technology, or fermentation vessel, for both red and white wine and the nearly exclusive use of oak is regulated by OIV rules and EU regulations; however, other non-oak species including cherry, chestnut, false acacia, and ash have also been applied beyond the EU (180, 181). The literature suggests that while oak lends most meaningfully to sensorial and organoleptic appeal, polyphenols and lignin derivatives are significantly more concentrated in ash, chestnut and cherry, where they are imparted on wine in a much shorter timeframe, as compared with oak (181). Similar to lignans, lignins, a complex, non-carbohydrate aromatic polymer found in all woods, continue to be a heavily researched area with high potential for applications in the treatment of obesity, cancer, thrombosis, viral infections and diabetes (Figure 2) (59, 179, 182). Research on oak and other wood species constituents and wine aging processes supports the transmission of additional beneficial compounds into finished wine.

## Conclusions

While cultivar contributes meaningfully to the level of anthocyanins and PA in wines, the influence of other viticulture practices is not insignificant. We hypothesize that wines produced from grapes cultivated between steady daily temperatures at 15–25°C with moderate sun exposure from flowering to harvest, lower vine-water status, resulting either from lower precipitation, and irrigation practices or more permeable soil types, will produce a wine with a higher concentration of anthocyanins and PA. The limitation of fertilizers in a soil rich with mineral deposits can also be considered in order to activate abiotic stress response. Furthermore, winemaker manipulation including maceration of red wines for 3 to 4 weeks appears to result in the greatest optimization of overall healthy phenols. Fining practices should be avoided or limited in use given their effect of decreasing phenolic compounds. Aging wine in oak may enhance tannin content along with lignan, lignin and other potentially healthy polyphenol content, however, wines aged excessively may experience a breakdown of anthocyanins. Wines consumed that follow these criteria are hypothesized to have a different, beneficial effect upon health.

All studies that have assessed the associations between wine consumption and health have viewed wine as a single entity. The above information provides significant evidence that wine consumption should not be viewed as a binary entity, and the multitude of growing and production conditions, including temperature, water availability, soil type, maceration, and aging can create a remarkably dissimilar final product. Based on the available studies, two types of wine with vastly different chemical and macronutrient profiles can be produced based on the type of grape, growing conditions, and production methods, and differing health results are to be expected after the consumption of these wines. Future studies should incorporate these differences.

## Author Contributions

Both CC and AK-C contributed in the design, data gathering, data synthesis, writing, and editing of the manuscript.

### Conflict of Interest Statement

CC receives compensation for nutrition books. The remaining author declares that the research was conducted in the absence of any commercial or financial relationships that could be construed as a potential conflict of interest.
